# Cell-based interferon gene therapy using proliferation-controllable, interferon-releasing mesenchymal stem cells

**DOI:** 10.1038/s41598-019-55269-6

**Published:** 2019-12-11

**Authors:** Mari Tsujimura, Kosuke Kusamori, Hidemasa Katsumi, Toshiyasu Sakane, Akira Yamamoto, Makiya Nishikawa

**Affiliations:** 1 Laboratory of Biopharmaceutics, Faculty of Pharmaceutical Sciences, Tokyo University of Science, 2641 Yamazaki, Noda, Chiba 278-8510 Japan; 2 Department of Biopharmaceutics, Kyoto Pharmaceutical University, 5 Nakauchi-cho, Misasagi, Yamashina-ku, Kyoto 607-8414 Japan

**Keywords:** Cancer therapy, Mesenchymal stem cells, Gene delivery, Genetic engineering

## Abstract

An important safety concern on cell-based gene therapy is that few methods have been available to control the proliferation and functioning of therapeutic protein-expressing cells after transplantation. We previously reported that the proliferation and functioning of the cells transfected with herpes simplex virus thymidine kinase (HSVtk) gene, a suicide gene, can be controlled by administration of ganciclovir. In this study, we tried to control the amount of murine interferon-γ (IFN-γ) secreted from transplanted murine mesenchymal stem cell line C3H10T1/2 cells to achieve safe cell-based IFN-γ gene therapy for cancer. C3H10T1/2 cells were transfected with HSVtk- and murine IFN-γ-expressing plasmid vectors to obtain C3H10T1/2/HSVtk/IFN-γ cells. C3H10T1/2/HSVtk/IFN-γ cells released IFN-γ and were sensitive to ganciclovir. C3H10T1/2/HSVtk/IFN-γ cells significantly suppressed the proliferation of murine adenocarcinoma cell line colon26 cells both *in vitro* and *in vivo*. Moreover, subcutaneous administration of ganciclovir to mice transplanted with NanoLuc luciferase-expressing C3H10T1/2/HSVtk cells for three consecutive days reduced the luminescence signals from the transplanted cells. These results indicate that the cell regulation system using HSVtk gene and ganciclovir can be useful for safe and efficient cell-based IFN-γ gene therapy for cancer.

## Introduction

Gene therapy for cancer is designed to transfer a gene encoding an antitumor protein or to transplant cells expressing such a gene to the patients^1,2^. This is an attractive therapeutic method as it can sustainably supply an antitumor protein for a long period with single gene transfer or cell transplantation. Unlike repeated administration in protein replacement therapy, this method can provide the stable and sustainable protein supply in the body. The first *in vivo* gene therapy was applied against the adenosine deaminase deficient patients in 1990. Since then, the therapeutic effects of *in vivo* gene therapy have been demonstrated for various diseases, e.g., Parkinson’s disease, Alzheimer’s disease, and cancers until now^3–6^. However, *in vivo* gene therapy has some crucial disadvantages to be solved. One of the most serious disadvantages is an unregulated protein synthesis because of gene overexpression, which can cause adverse effects. However, it is difficult to regulate the gene expression and protein synthesis in the body after gene transfer. Another disadvantage is serious immune responses, such as anaphylactic shock against the administered genes^7,8^.

Cell-based gene therapy, which is a therapeutic method to transplant genetically modified cells into patients, is another way that can sustainably supply a specific protein by single transplantation^9^. Studeny *et al*. showed that transplantation of human mesenchymal stem cells transduced with interferon-β gene into tumor-bearing mice resulted in a high plasma concentration of interferon-β and the excellent antitumor effects, compared to the injection of recombinant interferon-β protein^10^. Moreover, cell-based gene therapy does not need to directly transduce the gene into the body, and this reduces the risk of anaphylactic shock. However, even in cell-based gene therapy, it is difficult to regulate the amount of proteins secreted from the transplanted cells, as few methods have been developed to regulate the proliferation or functioning of the cells after transplantation^11^. Because the unregulated protein release from the transplanted cells may cause adverse effects, it is necessary to regulate the proliferation and/or functioning of the transplanted cells to achieve a safe cell-based gene therapy.

We previously developed a cell regulation system using cell suicide, which is the programmed cell death by apoptosis inducer, to control the proliferation and functioning of transplanted cells^12^. Cell suicide can cause the apoptosis of suicide gene-expressing cells in response to specific molecules, and we demonstrated that the proliferation and functioning of cells transfected with the herpes simplex thymidine kinase (HSVtk) gene, one of the suicide genes, could be regulated by administration of ganciclovir (GCV), a prodrug converted by HSVtk.

In this study, we tried to develop a safe cell-based interferon-γ (IFN-γ) gene therapy for cancer, by controlling the proliferation and functioning of the transplanted cells using the cell regulation system consisting of HSVtk gene and GCV. It has been demonstrated that IFN-γ has high antitumor activity in clinical use, and that the much higher therapeutic effects could be achieved by transplantation of IFN-γ-expressing cells into tumor-bearing mice, in some preclinical studies^13^. We first transfected the murine mesenchymal stem cell line C3H10T1/2 cells with HSVtk and IFN-γ genes to obtain the C3H10T1/2/HSVtk/IFN-γ cells. Then, we evaluated whether C3H10T1/2/HSVtk/IFN-γ cells could demonstrate antitumor effects against cultured cancer cells and in tumor-bearing mice. Furthermore, we also examined whether the proliferation of transplanted C3H10T1/2/HSVtk/IFN-γ cells could be regulated in mice by GCV administration, using C3H10T1/2/HSVtk cells expressing a reporter protein, NanoLuc luciferase (Nluc), for *in vivo* detection of the cells.

## Results

### Characteristics of C3H10T1/2/HSVtk/IFN-γ cells

**Figure 1:**
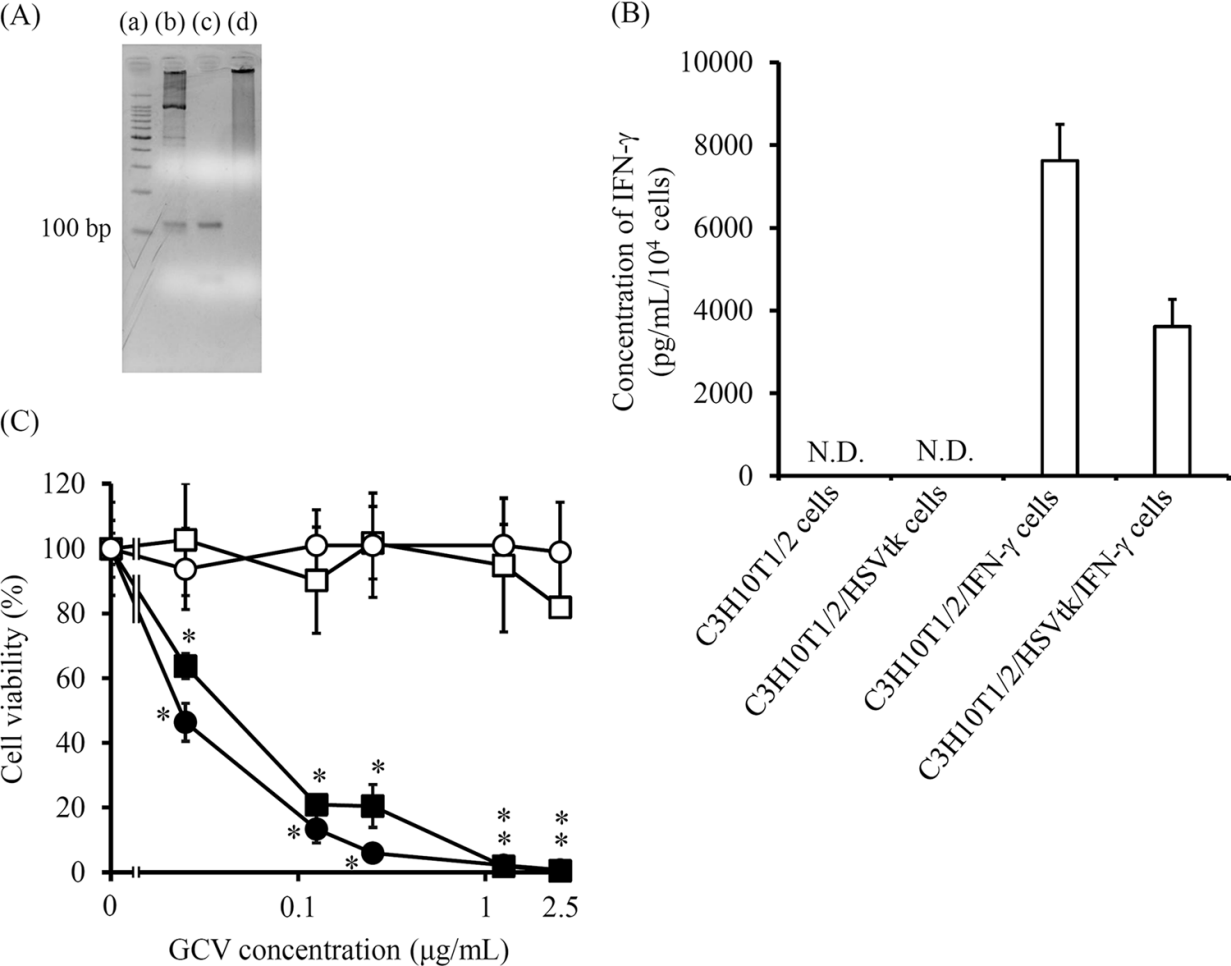
Characteristics of C3H10T1/2/HSVtk/IFN-γ cells. (**A**) The HSVtk-specific bands of PCR products on agarose gel after electrophoresis. The 100 bp DNA ladder (lane a), pCMV-HSVtk plasmid (lane b), C3H10T1/2/HSVtk cells (lane c), and C3H10T1/2 cells (lane d) are shown. (**B**) IFN-γ secretion of C3H10T1/2/IFN-γ and C3H10T1/2/HSVtk/IFN-γ cells. Cells were cultured for 24 h and the supernatants were collected. The concentration of IFN-γ in the supernatant was measured by ELISA. Results are expressed as the mean ± SD of four samples. A representative of four independent experiments with similar results is shown. (**C**) The viability of C3H10T1/2/HSVtk or C3H10T1/2/HSVtk/IFN-γ cells cultured with GCV at various concentrations. These cells were cultured in medium containing various concentration of GCV for four days. C3H10T1/2 cells (white circle), C3H10T1/2/IFN-γ cells (white square), C3H10T1/2/HSVtk cells (black circle), and C3H10T1/2/HSVtk/IFN-γ cells (black square) are indicated. Results are expressed as the mean ± SD of three to four samples. *p < 0.05; statistically significant differences observed in comparison with C3H10T1/2 cells group.

C3H10T1/2 cells transfected with pCMV-HSVtk plasmid were selected with G418 and cloned. The cells with the highest GCV sensitivity were used for further experiments. C3H10T1/2/IFN-γ or C3H10T1/2/HSVtk/IFN-γ cells were established by transfection of C3H10T1/2 or C3H10T1/2/HSVtk cells with pEBM-IFN-γ plasmid, followed by the selection with hygromycin. Figure 1 shows the characteristics of the established C3H10T1/2/HSVtk/IFN-γ cells. To confirm the HSVtk gene specific DNA in C3H10T1/2/HSVtk cells, cDNA from cells was amplified by PCR using HSVtk specific primers. Bands of HSVtk-specific PCR products were detected in the pCMV-HSVtk plasmid (Fig. 1A, lane b) and C3H10T1/2/HSVtk cells (Fig. 1A, lane c), but not in the C3H10T1/2 cells (Fig. 1A, lane d). To confirm the expression of IFN-γ gene in these cells, the concentration of IFN-γ in the culture media of C3H10T1/2/IFN-γ and C3H10T1/2/HSVtk/IFN-γ cells was measured. C3H10T1/2/IFN-γ and C3H10T1/2/HSVtk/IFN-γ cells released a large amount of IFN-γ (Fig. 1B). When C3H10T1/2, C3H10T1/2/HSVtk, C3H10T1/2/IFN-γ and C3H10T1/2/HSVtk/IFN-γ cells were cultured with medium containing GCV for 4 days, the viability of C3H10T1/2/HSVtk and C3H10T1/2/HSVtk/IFN-γ cells decreased with an increasing concentration of GCV, while that of C3H10T1/2 and C3H10T1/2/IFN-γ cells did not change (Fig. 1C).

### Effects of C3H10T1/2/IFN-γ or C3H10T1/2/HSVtk/IFN-γ cells on the proliferation of colon26/luc cells

**Figure 2:**
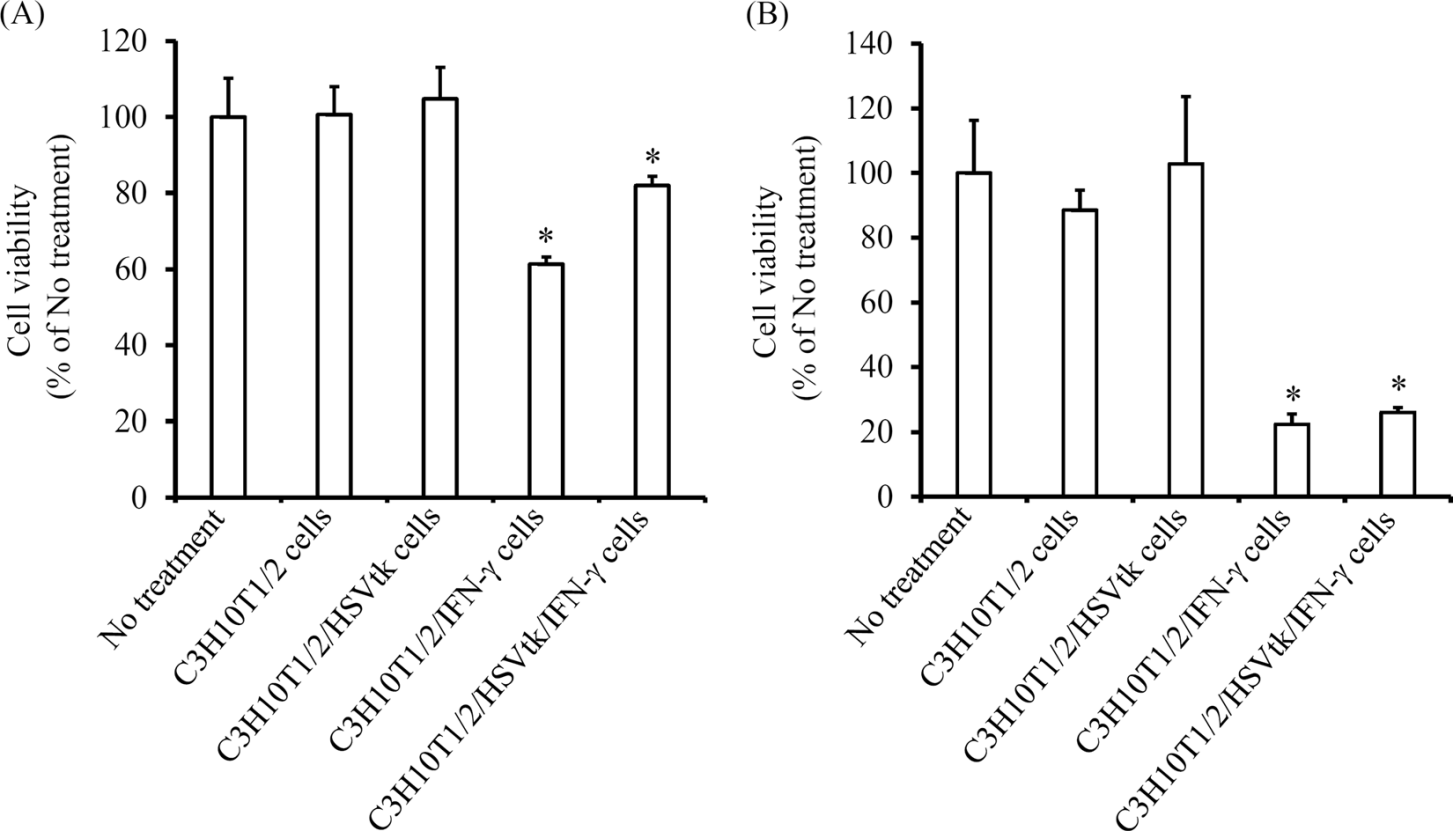
Effect of C3H10T1/2/IFN-γ cells or C3H10T1/2/HSVtk/IFN-γ cells on the proliferation of colon26/luc cells. (**A**) Viability of colon26/luc cells on incubation with the conditioned medium of C3H10T1/2/IFN-γ or C3H10T1/2/HSVtk/IFN-γ cells. The conditioned media of C3H10T1/2/IFN-γ or C3H10T1/2/HSVtk/IFN-γ cells were collected after 48 h incubation. The medium of colon26/luc cells was replaced with the conditioned media and the cells were cultured for 48 h. The viability of colon26/luc cells was calculated using CCK-8. Results are expressed as the mean ± SD of four samples. A representative of four independent experiments with similar results is shown. *p < 0.05; statistically significant differences observed in comparison with no treatment group. (**B**) Viability of colon26/luc cells after co-culture of C3H10T1/2/IFN-γ or C3H10T1/2/HSVtk/IFN-γ cells with colon26/luc cells. C3H10T1/2/IFN-γ or C3H10T1/2/HSVtk/IFN-γ cells and colon26/luc cells were co-cultured for 48 h. The viability of colon26/luc cells was calculated by detecting the luminescence of colon26/luc cells. Results are expressed as the mean ± SD of four samples. A representative of ten independent experiments with similar results is shown. *p < 0.05; statistically significant differences observed in comparison with no treatment group.

To evaluate the effects of cell-derived IFN-γ on the viability of cancer cells, C3H10T1/2, C3H10T1/2/HSVtk, C3H10T1/2/IFN-γ, or C3H10T1/2/HSVtk/IFN-γ cells were cultured for 48 h, and the supernatants were collected as conditioned medium. These conditioned media were added to the firefly luciferase gene-expressing murine adenocarcinoma cell line colon26/luc cells and cultured for 48 h. The conditioned medium of C3H10T1/2 or C3H10T1/2/HSVtk cells hardly affected the viability of colon26/luc cells (Fig. 2A). On the other hand, the conditioned medium of C3H10T1/2/IFN-γ and C3H10T1/2/HSVtk/IFN-γ cells significantly reduced the viability of colon26/luc cells. Furthermore, C3H10T1/2, C3H10T1/2/HSVtk, C3H10T1/2/IFN-γ or C3H10T1/2/HSVtk/IFN-γ cells were co-cultured with colon26/luc cells for 48 h. It was observed that C3H10T1/2 or C3H10T1/2/HSVtk cells hardly affected the viability of colon26/luc cells, while C3H10T1/2/IFN-γ and C3H10T1/2/HSVtk/IFN-γ cells significantly reduced the viability of these cells (Fig. 2B).

### Effect of C3H10T1/2/IFN-γ or C3H10T1/2/HSVtk/IFN-γ cells on colon26/luc-tumor growth in mice

**Figure 3:**
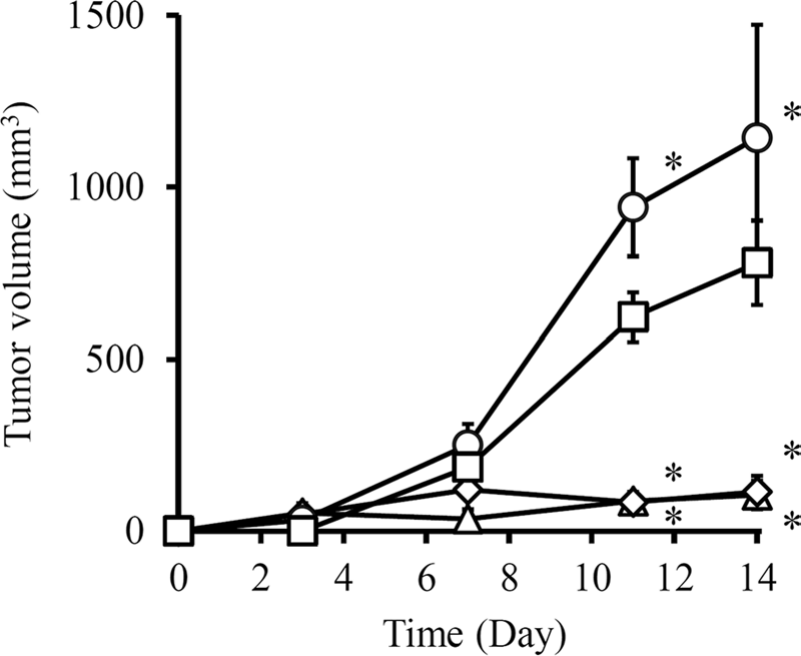
Effect of C3H10T1/2/IFN-γ or C3H10T1/2/HSVtk/IFN-γ cells on tumor growth. Colon26/luc cells were mixed with C3H10T1/2/IFN-γ or C3H10T1/2/HSVtk/IFN-γ cells and transplanted into the back of BALB/c Slc-*nu/nu* mice. The tumor volume was measured by twice a week using a caliper. Colon26/luc cells (white square), colon26/luc cells and C3H10T1/2 cells (white circle), colon26/luc cells and C3H10T1/2/IFN-γ cells (white diamond), and colon26/luc cells and C3H10T1/2/HSVtk/IFN-γ cells (white triangle) are indicated. Results are expressed as the mean ± SD of five mice. A representative of two independent experiments with similar results is shown. *p < 0.05; statistically significant differences observed in comparison with colon26/luc cells group.

To confirm the effect of IFN-γ-expressing cells on tumor growth in mice, colon26/luc cells were mixed with C3H10T1/2, C3H10T1/2/IFN-γ or C3H10T1/2/HSVtk/IFN-γ cells, and transplanted into the back of mice. Figure 3 shows the time course of the tumor volume in mice after transplantation of colon26/luc cells. Co-administration of C3H10T1/2 cells significantly increased the colon26/luc tumor volume in mice. On the other hand, co-administration of C3H10T1/2/IFN-γ or C3H10T1/2/HSVtk/IFN-γ cells almost completely suppressed the tumor growth. In addition, C3H10T1/2/HSVtk/IFN-γ cells significantly suppressed the tumor growth in mice with metastatic lung cancer of B16-Bl6/Nluc cells compared with C3H10T1/2 cells (Supplementary Fig. 1).

### Effect of GCV on the proliferation of C3H10T1/2/HSVtk or C3H10T1/2/HSVtk/IFN-γ cells

**Figure 4:**
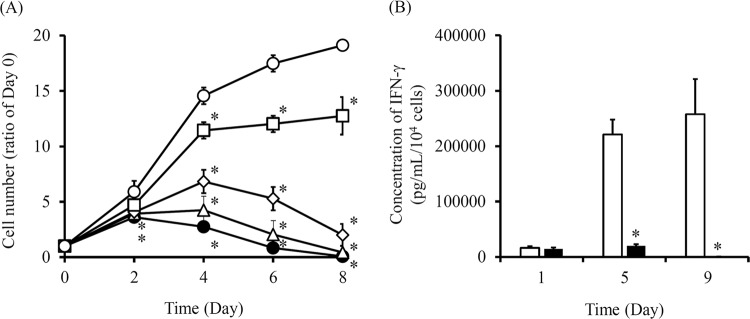
Effect of GCV on the proliferation of C3H10T1/2/IFN-γ or C3H10T1/2/HSVtk/IFN-γ cells. (**A**) The number of C3H10T1/2/HSVtk cells treated with GCV. C3H10T1/2/HSVtk cells were cultured in media containing 0.03125, 0.0625, 0.125 or 0.25 μg/mL GCV. No treatment (white circle), 0.03125 μg/mL GCV (white square), 0.0625 μg/mL GCV (white diamond), 0.125 μg/mL GCV (white triangle), and 0.25 μg/mL GCV (black circle) are indicated. Results are expressed as the mean ± SD of four samples. A representative of two independent experiments with similar results is shown. *p < 0.05; statistically significant differences observed in comparison with no treatment group. (**B**) The IFN-γ concentration in the supernatant of C3H10T1/2/HSVtk/IFN-γ cells cultured in normal medium or GCV containing medium. C3H10T1/2/HSVtk/IFN-γ cells were cultured in medium containing 0.25 μg/mL GCV. The medium was replaced with fresh medium containing various concentrations of GCV, and it was changed every 24 h and collected at Day 5 and Day 9. The IFN-γ concentration in medium was measured by ELISA. No treatment (white bar) and 0.25 μg/mL GCV containing medium (black bar) are indicated. Results are expressed as the mean ± SD of four samples. A representative of two independent experiments with similar results is shown. *p < 0.05; statistically significant differences observed in comparison with no treatment group.

To examine the change in the proliferation and functioning of HSVtk/IFN-γ expressing cells by GCV, C3H10T1/2/HSVtk cells were cultured in media containing various concentrations of GCV, and the number of cells was measured every two days (Fig. 4A). The cell number ratio was calculated by setting the cell number of Day 0 as 1. The number of C3H10T1/2/HSVtk cells cultured in normal medium without GCV increased with time. On the other hands, the number of C3H10T1/2/HSVtk cells cultured in GCV containing medium decreased in a GCV concentration-dependent manner. Similarly, C3H10T1/2/HSVtk/IFN-γ cells were cultured in normal medium or media containing various concentration of GCV, and the IFN-γ concentration in the supernatant was measured (Fig. 4B). The IFN-γ concentration of C3H10T1/2/HSVtk/IFN-γ cells in normal medium increased with time. Conversely, IFN-γ concentration of C3H10T1/2/HSVtk/IFN-γ cells cultured in 0.25 μg/mL GCV containing medium was not detected by Day 8.

### Elimination of C3H10T1/2/HSVtk/Nluc cells from mice by GCV

**Figure 5:**
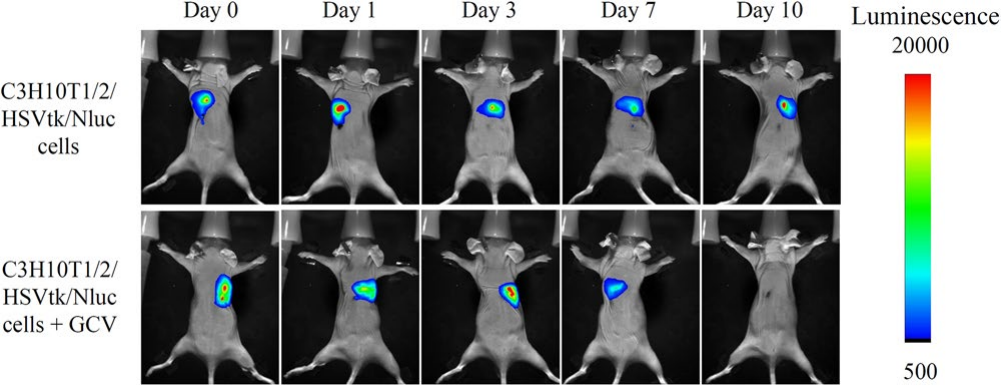
Elimination of C3H10T1/2/HSVtk/Nluc cells from mice by GCV. C3H10T1/2/HSVtk/Nluc cells were subcutaneously transplanted into the back of BALB/c Slc-*nu/nu* mice. GCV (50 mg/kg) was subcutaneously administered into mice for three consecutive days from Day 7 after cell transplantation. The luminescence of cells transplanted in mice was detected in an *In-Vivo* Xtreme Imaging System.

Figure 5 shows the luminescent images of mice transplanted with C3H10T1/2/HSVtk/Nluc cells before and after GCV administration. First, we confirmed that the transplanted C3H10T1/2/HSVtk/Nluc cells into mice survived over four weeks (Supplementary Fig. 2). Then, C3H10T1/2/HSVtk/Nluc cells were transplanted into the back of mice, and 50 mg/kg GCV was subcutaneously administered for three consecutive days, from Day 7 after cell transplantation. In a previous report, we showed that the survival of cells without HSVtk gene was hardly affected by GCV administration^12^. Without GCV administration, the luminescent signal derived from C3H10T1/2/HSVtk/Nluc cells was detected in mice until Day 10 after transplantation. On the other hand, daily administration of 50 mg/kg GCV from Day 7 to Day 10 extinguished the luminescent signal from the cells. Furthermore, we also evaluated the IFN-γ amount in the skin of C3H10T1/2/HSVtk/IFN-γ cells-transplanted mice and luciferase activity in the skin of C3H10T1/2/Nluc/HSVtk cells-transplanted mice with or without GCV administration (Supplementary Fig. 3). The IFN-γ concentration in the skin of the C3H10T1/2/HSVtk/IFN-γ cell-transplanted, GCV-treated mice was significantly lower than that of the C3H10T1/2/HSVtk/IFN-γ cell-transplanted, GCV-untreated mice. The IFN-γ concentration in the skin of the C3H10T1/2/HSVtk/IFN-γ cell-transplanted mice was almost the same as that of the no treatment mice (42 ± 33 pg/mL). Similarly, the luciferase activity in the skin of the C3H10T1/2/Nluc/HSVtk cell-transplanted, GCV-treated mice was significantly lower than that of the C3H10T1/2/Nluc/HSVtk cell-transplanted, GCV-untreated mice.

### Evaluation of the level of creatinine, BUN, AST and ALT in plasma and body weight of mice after GCV administration

Repeated dosing of GCV (50 mg/kg, twice a day) for 10 days to C3H10T1/2/Nluc/HSVtk cells-transplanted mice hardly affected the plasma levels of creatinine, BUN, AST and ALT. In addition, the body weight of the mice was hardly changed by GCV administration (Supplementary Fig. 4).

## Discussion

Many protein pharmaceutical products including IFN are clinically used in the treatment of various diseases and their effectiveness has also been confirmed^14^. However, the administration of these products by injection can be painful to the patients, and some products may require repeated administration because of their short *in vivo* half-life^15^. Thus, the patient’s quality of life is generally low. To reduce the dose frequency, pegylated proteins such as peginterferon and pegfilgrastim have been developed^16,17^. However, the activities of proteins may decrease in some cases, because PEG modification may reduce the binding affinity of proteins with receptors or may change the structure of proteins^18^. Hence, we thought that gene therapy, especially cell-based gene therapy, could solve the problem of protein pharmaceutical products. In this study, we first succeeded in the regulation of functioning of cell-based gene therapy using cell regulation system consisting of HSVtk gene and GCV^12^. When the adverse effects occur in patients because of the excessive protein secretion in cell-based gene therapy, the concentration of the therapeutic protein can be adequately regulated by the administration of GCV. Therefore, this cell regulation system is a mandatory step for the safe cell-based gene therapy.

Cell-based gene therapy for cancer has demonstrated an excellent antitumor effect in many reports. For example, TRAIL, interleukin (IL)-12, IL-27, cytosine deaminase, and oncolytic adenovirus are transduced into MSC to treat cancer. Therefore, the cell-based gene therapy can be applied to various types of cancers after selecting suitable anti-tumor cytokines^13,19–25^. We selected cancer and IFN-γ as the disease and the therapeutic molecule to demonstrate the usefulness of cell regulation system in cell-based gene therapy in this study. In addition, we used mesenchymal stem cell line C3H10T1/2 cells, because mesenchymal stem cells have been clinically used for many diseases in recent years^26–29^. Moreover, mesenchymal stem cells, among the other stem cells, are reported to rarely cause tumorigenesis^30,31^. Therefore, mesenchymal stem cells are safe for transplantation, and can be appropriate cells for cell-based gene therapy.

In Fig. 2A, colon26/luc cells showed reduced viability when cultured in the conditioned medium of C3H10T1/2/IFN-γ, compared to the conditioned medium of C3H10T1/2/HSVtk/IFN-γ cells. This is because the IFN-γ secretion from C3H10T1/2/IFN-γ cells was higher than that of C3H10T1/2/HSVtk/IFN-γ cells, suggesting that the cell viability is dependent on the concentration of IFN-γ. On the other hand, in co-culture experiments, the viability of colon26/luc cells was almost the same for C3H10T1/2/IFN-γ and C3H10T1/2/HSVtk/IFN-γ cells (Fig. 2B). This may be because co-culture of C3H10T1/2/IFN-γ or C3H10T1/2/HSVtk/IFN-γ cells with colon26/luc cells had the higher antitumor effect than the conditioned medium. Probably because of effective delivery of IFN-γ in short distances between the cells, the different concentration of IFN-γ may have little effects on the viability of colon26/luc cells under the experimental conditions. Similar results were obtained in the *in vivo* antitumor experiment (Fig. 3).

Mesenchymal stem cells have been reported to show tumor supportive effects or tumor suppressive effects in some reports^32^. The tumor supportive effects of mesenchymal stem cells are caused by the suppression of immune response by secretion of immunosuppressive cytokines^33–35^. In contrast, the proliferation of malignant glioma cell lines was significantly inhibited by the treatment with human umbilical cord blood mesenchymal stem cells *in vitro* and *in vivo*. This is because the mesenchymal stem cells induced apoptosis in glioma cells by inhibition of the inhibitor of apoptosis protein^36^. Our results showed that co-injection of C3H10T1/2 cells increased the growth of colon26/luc tumor, suggesting that C3H10T1/2 cells may have tumor supportive effects. Further usefulness of C3H10T1/2/HSVtk/IFN-γ cells needs to be examined in tumor-bearing mice in future studies.

The apoptosis induction of HSVtk-expressing cells by GCV needs a few days, because the inhibition of DNA synthesis by phosphorylated GCV requires some time to induce cell apoptosis as previously reported^37,38^. Our result showed 0.25 μg/mL GCV significantly suppressed the cell proliferation on Day 4 compared to untreated cells, and completely eliminated the cells on Day 8 (Fig. 4A). The results were consistent with the IFN-γ secretion in C3H10T1/2/HSVtk/IFN-γ cells (Fig. 4B). Similarly, our previous study showed that HSVtk-expressing mouse pancreatic beta cell line MIN6 (MIN6/HSVtk) cells also needed a few days to completely undergo apoptotic cell death by GCV^12^. It is reported that the required time of apoptosis induction in cell suicide varies depending on the type of suicide genes^39^. Because the number of transplanted cells in cell-based gene therapy directly correlates with the therapeutic protein level in the systemic circulation, the type of suicide gene, i.e., the required time of cell apoptosis, should be selected depending on the disease. In addition, the amount of GCV for cell apoptosis induction is important to regulate the cell proliferation. Figure 5 shows that transplanted C3H10T1/2/HSVtk/Nluc cells in mice disappeared by administration of 50 mg/kg GCV for three consecutive days. Our previous study showed that 100 mg/kg GCV administration completely removed the MIN6/HSVtk/Nluc cells from mice, whereas the cell number remained constant after daily 50 mg/kg GCV administration^12^. From these results, the required amount of GCV for apoptosis induction might depend on the expression level of HSVtk gene or the type of cells. Further studies are needed to estimate the required amount of GCV for the *in vivo* cell regulation.

In conclusion, cell-based IFN-γ gene therapy for cancer with the cell regulation system using HSVtk gene and GCV modulated the amount of IFN-γ secreted from genetically modified cells. Furthermore, the transplanted cells could be removed from the mice by GCV administration, indicating that this cell regulation system is essential for the safe and efficient cell-based IFN-γ gene therapy for cancer.

## Materials and Methods

### Animals

Male BALB/c Slc-*nu/nu* mice (5 weeks old) were purchased from Japan SLC, Inc. (Shizuoka, Japan) and maintained under specific pathogen-free conditions. The protocols for experiments involving animals were approved by the Institutional Animal Experimentation Committee of Kyoto Pharmaceutical University and Tokyo University of Science. All experiments involving animals were conducted in accordance with the procedures outlined in the National Instituted of Health Guide for the Care and Use of Laboratory Animals.

### Materials

The pSelect-zeo-HSV1tk plasmid, G418, Hygromycin B Gold, LB Broth Base, and LB Agar were purchased from InvivoGen Co. (San Diego, CA, USA). Lipofectamine 3000 was purchased from Invitrogen Co. (Carlsbad, CA, USA). pLVSIN-CMV-Neo plasmid, and Lentiviral High Titer Packaging Mix were purchased from Takara Bio Inc. (Shiga, Japan). KOD-Plus-Neo was purchased from TOYOBO Co., Ltd. (Osaka, Japan). Competent NEB 10-beta *E. coli* (High efficiency), SOC medium, *KpnI*-HF, *EcoRI*-HF, and *NotI*-HF were purchased from New England Biolabs Inc. (Ipswich, MA, USA). Ganciclovir hydrate was purchased from Tokyo Kasei Kogyo Co., Ltd. (Tokyo, Japan). Hank’s Balanced Salt solution was purchased from Sigma-Aldrich (St. Louis, MO, USA). Chloroform, isopropyl alcohol, ethanol, NaHCO_3_, D-(+)-glucose, 0.4 w/v% trypan blue solution, and pEBMulti-Hyg plasmid were purchased from Wako Pure Chemical Industries, Ltd. (Osaka, Japan). Fetal bovine serum was purchased from Biosera (East Sussex, UK) or Thermo Fisher Scientific (Waltham, MA, USA). Dulbecco’s Modified Eagle’s medium (DMEM), antibiotic-antimycotic mixed stock solution, and penicillin-streptomycin-glutamine mixed solution were purchased from Nacalai Tesque, Inc. (Kyoto, Japan) or Nissui Seiyaku (Tokyo, Japan). All the other chemicals used were of the highest grade available commercially.

### Cell culture

Dr. Hiroki Kagawa (Department of Cell Biology, Kyoto Pharmaceutical University, Kyoto, Japan) kindly provided the murine mesenchymal stem cell line C3H10T1/2 cells. C3H10T1/2 cells were cultured in DMEM supplemented with 15% heat-inactivated fetal bovine serum and antibiotic-antimycotic mixed stock solution or penicillin-streptomycin-glutamine mixed solution at 37 °C in humidified air containing 5% CO_2_. Colon26 cells (colon26/luc cells) were cultured in RPMI supplemented with 10% heat-inactivated fetal bovine serum and antibiotic-antimycotic mixed stock solution or penicillin-streptomycin-glutamine mixed solution at 37 °C in humidified air containing 5% CO_2_. Lenti-X 293 T cells were purchased from Takara Bio Inc. (Shiga, Japan) and cultured in DMEM supplemented with 10% heat-inactivated FBS, penicillin-streptomycin-glutamine mixed solution, and 1 mM Sodium Pyruvate Solution (100×) at 37 °C in humidified air containing 5% CO_2_. NanoLuc Luciferase-expressing mouse melanoma B16-BL6 cells (B16-Bl6/Nluc cells) were cultured in DMEM supplemented with 10% heat-inactivated fetal bovine serum and penicillin-streptomycin-glutamine mixed solution at 37 °C in humidified air containing 5% CO_2_.

### Construction of plasmids

To construct a plasmid coding IFN-γ gene (pEBMulti-IFN-γ), the fragment of IFN-γ gene was amplified by polymerase chain reaction (PCR) using primers (forward: 5′-CGGGGTACCATGAACGCTACACACT-3′ and reverse: 5′- TAAAGCGGCCGCTCAGCAGCGACT-3′). Subsequently, the amplified PCR product and pEBMulti-hyg vector were digested using *KpnI*-HF and *NotI*-HF restriction enzymes overnight at 37 °C. Ligation and cloning in *E. coli* was performed as previously reported^12^. Separately, to construct an HSVtk-encoding lentiviral plasmid, pLVSIN-HSVtk, the cDNA fragment of the HSVtk gene was amplified by PCR using primers (forward: 5′-CCGGAATTCATGGCTTCTTACCCTG-3′ and reverse: 5′-TAAAGCGGCCGCTTAGTTGGCCTCT-3′) from the pSelect-zeo-HSV1tk plasmid. Subsequently, the amplified PCR product and pLVSIN-CMV-Neo plasmid were digested using EcoRI-HF and NotI-HF restriction enzymes overnight at 37 °C. Ligation and cloning were performed as described above. pCMV-HSVtk and pEBMulti-NanoLuc luciferase (Nluc) plasmids were prepared in our previous study^12^.

### Establishment of the gene-expressing C3H10T1/2 cells

HSVtk-expressing C3H10T1/2 cells were established as previously reported^12^. Briefly, C3H10T1/2 cells (1 × 10^5^ cells) were seeded in a 24-well culture plate and cultured overnight at 37 °C in humidified air containing 5% CO_2_. C3H10T1/2 cells were transfected with pCMV-HSVtk plasmid using Lipofectamine 3000 in Opti-MEM. After 24 h, the medium was replaced and cultured in a normal culture medium for a day. Then, these cells were cultured in medium containing 1500 μg/mL G418 for 5 days, and cloned and incubated until they were confluent. Cloned cells, which showed the induction of apoptosis by GCV were selected as the C3H10T1/2/HSVtk cells. To establish IFN-γ or Nluc-expressing C3H10T1/2 (C3H10T1/2/IFN-γ or C3H10T1/2/Nluc) cells and IFN-γ or Nluc-expressing C3H10T1/2/HSVtk (C3H10T1/2/HSVtk/IFN-γ or C3H10T1/2/HSVtk/Nluc) cells, C3H10T1/2 cells or C3H10T1/2/HSVtk cells (1 × 10^5^ cells) were seeded in a 24-well culture plate and transfected with pEBMutli-IFN-γ plasmid or pEBMulti-Nluc plasmid using Lipofectamine 3000 as described above. After these cells were cultured in medium for a day, pEBMulti-IFN-γ or pEBMulti-Nluc-transfected C3H10T1/2 cells were cultured in medium containing 500 μg/mL hygromycin and pEBMulti-IFN-γ- or pEBMulti-Nluc-transfected C3H10T1/2/HSVtk cells were cultured in medium containing 300 μg/mL hygromycin. The expression of IFN-γ in cells was confirmed by measuring IFN-γ in the cell culture medium using an enzyme-linked immunosorbent assay (ELISA) kit (BioLegend, San Diego, CA, USA). The expression of Nluc in cells was confirmed by detecting the luminescence using a Nano-Glo assay reagent (Promega Co., Tokyo, Japan).

Lenti-X 293 T cells (1 × 10^7^ cells) were seeded in a 10 cm culture dish and cultured overnight at 37 °C in humidified air containing 5% CO_2_. Lenti-X 293 T cells were co-transfected with pLVSIN-HSVtk plasmid and Lentiviral High Titer Packaging Mix by the calcium phosphate method. After 24 h, the medium was replaced with culture medium containing 10 μM forskolin, and cells were cultured for an additional 48 h. Then, the supernatant was collected and centrifuged at 900 g for 5 min. Furthermore, the supernatant was filtered, mixed with PEG solution (32 w/v% polyethylene glycol #6000, 400 mM NaCl, and 40 mM HEPES), and centrifuged at 2,500 g for 40 min. The precipitated viruses were collected and suspended by Opti-MEM. Then, C3H10T1/2/Nluc cells (1 × 10^5^ cells) were seeded in a 12-well culture plate and transduced by the suspended virus. The medium was replaced after 24 h, and the cells were cultured in normal culture medium for three days, followed by 5-day culture in medium containing 1500 μg/mL G418. These cells were cloned and selected as C3H10T1/2/Nluc/HSVtk cells, which underwent apoptosis by GCV.

### Confirmation of the HSVtk gene-specific DNA

Detection of the HSVtk gene-specific DNA in cells was performed using the protocol of our previous research^12^. Briefly, RNA was extracted from C3H10T1/2 or C3H10T1/2/HSVtk cells using Sepasol RNA I Super G (Nacalai Tesque, Inc.). The obtained RNA was converted to cDNA using ReverTra Ace qPCR RT Master Mix with gDNA Remover (TOYOBO Co., Ltd.). The HSVtk-specific PCR fragment in each cDNA was amplified by PCR using HSVtk specific primers (forward: 5′-AACATCTACACCACCCAGCAC-3′ and reverse: 5′-GAACAGCATCAGTCACAGCATAG-3′). The PCR products were separated on a 3% agarose gel and stained with ethidium bromide. The stained bands on the agarose gel were detected using a LAS 4000 mini imaging system (Fujifilm Co., Ltd., Tokyo, Japan).

### IFN-γ secretion

C3H10T1/2/IFN-γ or C3H10T1/2/HSVtk/IFN-γ (1 × 10^5^ cells) cells were seeded in a 12-well culture plate and cultured for 24 h at 37 °C in humidified air containing 5% CO_2_. The culture media were collected and centrifuged at 800 × *g* for 3 min. The concentration of IFN-γ secreted from C3H10T1/2/IFN-γ or C3H10T1/2/HSVtk/IFN-γ cells in the supernatant was measured using an IFN-γ ELISA kit.

### Sensitivity of HSVtk-expressing C3H10T1/2 cells to GCV

C3H10T1/2, C3H10T1/2/HSVtk, C3H10T1/2/IFN-γ, and C3H10T1/2/HSVtk/IFN-γ cells (5 × 10^3^ cells) were seeded in a 12-well culture plate and cultured overnight at 37 °C in humidified air containing 5% CO_2_. Next day, the medium was replaced with fresh medium containing various concentrations of GCV. After four days, the number of viable cells was counted by using trypan blue exclusion test.

### Effects of C3H10T1/2/IFN-γ or C3H10T1/2/HSVtk/IFN-γ cells on the proliferation of colon26/luc cells

C3H10T1/2/IFN-γ or C3H10T1/2/HSVtk/IFN-γ cells (1 × 10^5^ cells) were seeded in a 12-well culture plate and cultured for 48 h at 37 °C in humidified air containing 5% CO_2_. The supernatants were collected and centrifuged at 800 × *g* for 3 min (conditioned medium). Colon26/luc cells (5 × 10^3^ cells) were seeded in a 96-well culture plate and cultured overnight at 37 °C in humidified air containing 5% CO_2_. Next day, the culture medium of colon26/luc cells was replaced with the conditioned medium of C3H10T1/2/IFN-γ or C3H10T1/2/HSVtk/IFN-γ. The cells were cultured for 48 h and the number of cells was measured by the Cell Counting Kit-8 (Dojindo Laboratories, Kumamoto, Japan).

Additionally, colon26/luc cells (2.5 × 10^4^ cells) were seeded in a 12-well culture plate and cultured overnight at 37 °C in humidified air containing 5% CO_2_. Next day, C3H10T1/2/IFN-γ or C3H10T1/2/HSVtk/IFN-γ cells (2 × 10^5^ cells) were co-cultured with colon26/luc cells for 48 h at 37 °C in humidified air containing 5% CO_2_. These cells were collected and lysed using lysis buffer (20 mM Tris-HCl, 200 mM NaCl, 2.5 mM MgCl_2_, 0.05 w/v% NP-40, pH 7.4). Total 20 μL of these cell lysates were mixed with 10 μL of Picagene kit (Toyo Inki, Tokyo, Japan) and the luminescence was measured with EnVision multi-label plate reader (Perkin-Elmer, Wellesley, MA, USA). The viability of colon26/luc cells was calculated as 100% of the luminescence of the colon26/luc cells.

### Effect of C3H10T1/2/IFN-γ or C3H10T1/2/HSVtk/IFN-γ cells on tumor growth

Colon26/luc cells (2.5 × 10^5^ cells) were mixed with or without C3H10T1/2 (2 × 10^6^ cells), C3H10T1/2/IFN-γ (2 × 10^6^ cells) or C3H10T1/2/HSVtk/IFN-γ cells (5 × 10^6^ cells) and transplanted into the back of BALB/c Slc-*nu/nu* mice. The tumor diameters in mice were measured by twice a week using a caliper. The tumor volumes were calculated from the following formula: (large diameter) × (small diameter)^2^ × 0.5236. B16-Bl6/Nluc cells (2.5 × 10^5^ cells) were intravenously inoculated, and C3H10T1/2 or C3H10T1/2/HSVtk/IFN-γ cells (5 × 10^6^ cells) were intravenously administrated 2 h after B16-Bl6/Nluc cell inoculation. After 7 days, the lungs were removed from mice and homogenized in a lysis buffer using a homogenizer. Then, the homogenate was centrifuged at 10,000 × *g*for 10 min at 4 °C. The luciferase activity in the supernatant was evaluated using a Nano-Glo assay reagent.

### Effect of GCV on the proliferation of C3H10T1/2/HSVtk cells

C3H10T1/2/HSVtk cells (1 × 10^3^ cells) were seeded in a 96-well culture plate and cultured overnight at 37 °C in humidified air containing 5% CO_2_, and the number of cells was measured by Cell Counting Kit-8 (CCK-8, Dojindo Laboratories). The cells were next cultured in medium containing various concentrations of GCV and the number of cells was repeatedly measured every 2 days by using CCK-8.

### Effect of GCV on the proliferation of C3H10T1/2/HSVtk/IFN-γ cells

C3H10T1/2/HSVtk/IFN-γ cells (5 × 10^4^ cells) were seeded in a 24-well culture plate and cultured overnight at 37 °C in humidified air containing 5% CO_2_, and the medium was collected and centrifuged at 800 × *g* for 3 min. The medium was replaced with fresh medium containing various concentrations of GCV, which was replaced every 24 h and collected at Day 5 and Day 9. The concentration of IFN-γ in the supernatant was measured using an IFN-γ ELISA kit.

### Evaluation of the survival of transplanted C3H10T1/2/HSVtk/Nluc cells in mice

C3H10T1/2/HSVtk/Nluc cells (1 × 10^6^ cells) were mixed with Matrigel and transplanted into the back of BALB/c Slc-*nu/nu* mice. The blood was collected from the facial vein with a heparinized tube and Goldnord animal lancet (MEDIpoint, Inc., Mineola, NY, USA) two or three times a week for four weeks.

### Transplantation of C3H10T1/2/HSVtk/Nluc cells and their elimination by GCV

C3H10T1/2/HSVtk/Nluc cells (1 × 10^6^ cells) were mixed with Matrigel (BD Biosciences, San Diego, CA, USA) to improve the survival of cells in transplantation. The cells were transplanted into the back of BALB/c Slc-*nu/nu* mice. Seven days after cell transplantation, 50 mg/kg GCV was subcutaneously administered to mice for three consecutive days. The luminescence of cells in mice was detected using an *In-Vivo* Xtreme Imaging System (Bruker, Billerica, MA, USA) after injection of Nano-Glo assay reagent into the site of cell transplantation at a dose of 50 μL/mouse.

### Measurement of IFN-γ and luciferase after inoculation of C3H10T1/2/HSVtk/IFN-γ cells and C3H10T1/2/Nluc/HSVtk cells, respectively, in mice skin

C3H10T1/2/HSVtk/IFN-γ cells (5 × 10^6^ cells) or C3H10T1/2/Nluc/HSVtk cells (1 × 10^6^ cells) were transplanted into the back of BALB/c Slc-*nu/nu* mice. GCV was subcutaneously administered at a dose of 50 mg/kg soon after transplantation and every 12 h subsequently for ten consecutive days. The skin tissue around the transplantation site of C3H10T1/2/HSVtk/IFN-γ cells was cut off 12 h after the last GCV administration and homogenized in a lysis buffer using a homogenizer (Microtec, Chiba, Japan). Then, the homogenate was centrifuged at 10,000 g for 10 min at 4 °C. The IFN-γ concentration in the supernatant was evaluated using an IFN-γ ELISA kit. Likely, the skin tissue around the cell transplantation site of C3H10T1/2/Nluc/HSVtk cells was cut off 36 h after the last GCV administration and homogenized as above. Then, the homogenate was centrifuged at 10,000 g for 10 min at 4 °C. The luciferase activity in the supernatant was evaluated using Nano-Glo assay reagent (Promega).

### Evaluation of the level of creatinine, BUN, AST and ALT in plasma, and body weight of mice after GCV administration

After 10 consecutive days of GCV administration (50 mg/kg, twice a day) to C3H10T1/2/Nluc/HSVtk cells-transplanted mice, blood was collected from the postcaval vein. The plasma was collected from the blood by centrifugation for 5 min at 13,000 × *g* at 4 °C. Creatinine, blood urea nitrogen (BUN), aspartate aminotransferase (AST) and alanine aminotransferase (ALT) activities in plasma were measured using Labassay Creatinine (FUJIFILM, Wako Pure Chemical Industries, Ltd., Osaka, Japan), QuantiChrom Urea Assay Kit (Hayward, CA, USA) and transaminase CII test Wako (FUJIFILM, Wako Pure Chemical Industries, Ltd., Osaka, Japan). In addition, the body weight of mice was measured every other day.

### Statistical analysis

Statistical differences were evaluated by one-way analysis of variance (ANOVA) followed by the Dunnett’s test for multiple comparisons or the Student’s t-test for two groups. p-values that were < 0.05 were considered statistically significant.

## Supplementary information

Supplementary

## Data Availability

All data used for this research are publicly available.
