# Supplementary material for: Cell-based interferon gene therapy using proliferation-controllable, interferon-releasing mesenchymal stem cells
Source: Sci Rep. 2019 Dec 11;9:18869. doi: 10.1038/s41598-019-55269-6 (PMC6906518; doi:10.1038/s41598-019-55269-6)
Supplement: Supplementary file 1 — Supplementary [file 41598_2019_55269_MOESM1_ESM.pdf]

Supplementary Information

Cell-based interferon gene therapy using proliferation-controllable, interferon-releasing mesenchymal stem cells

Mari Tsujimura[1], Kosuke Kusamori[1,*], Hidemasa Katsumi[2], Toshiyasu Sakane[2], Akira Yamamoto[2], and Makiya Nishikawa[1]

[1] Laboratory of Biopharmaceutics, Faculty of Pharmaceutical Sciences, Tokyo University of Science, 2641 Yamazaki, Noda, Chiba 278-8510, Japan

[2] Department of Biopharmaceutics, Kyoto Pharmaceutical University, 5 Nakauchi-cho, Misasagi, Yamashina-ku, Kyoto 607-8414, Japan

Supplementary. Fig. 1.

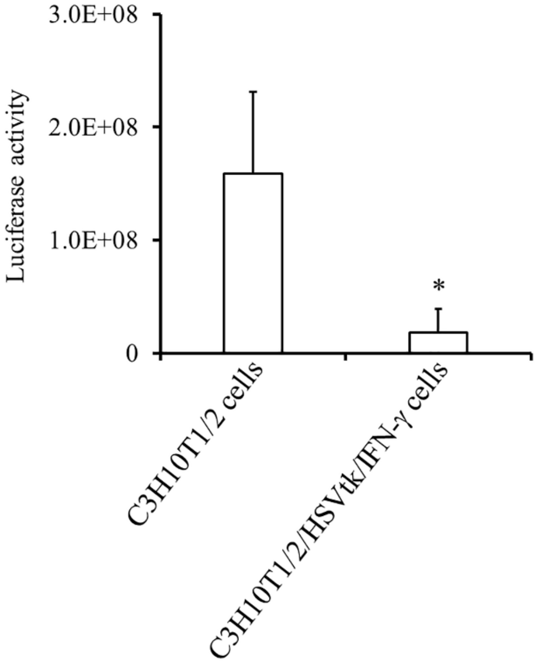

Supplementary. Fig. 1. Effect of C3H10T1/2 or C3H10T1/2/HSVtk/IFN-γ cells on the tumor growth in mice with metastatic lung cancer. B16-Bl6/Nluc cells were intravenously inoculated, and C3H10T1/2 or C3H10T1/2/HSVtk/IFN-γ cells were intravenously administered 2 h after B16-Bl6/Nluc cell inoculation. After 7 days, the lungs were removed from mice and the luciferase activity of lung was evaluated using a Nano-Glo assay reagent. Results are expressed as the mean ± SD of five to six samples. *$p < 0.05$, statistically significant difference observed in comparison with C3H10T1/2 cells administration group.

Supplementary. Fig. 2.

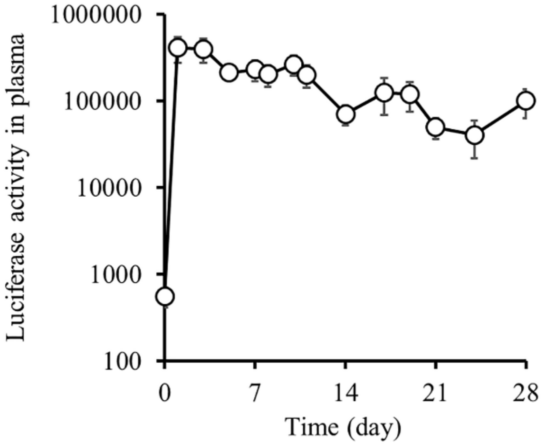

Supplementary. Fig. 2. The survival of transplanted C3H10T1/2/HSVtk/Nluc cell in mice. C3H10T1/2/HSVtk/Nluc cells with Matrigel were transplanted into the back of BALB/c Slc-*nu/nu* mice. The blood was collected from the facial vein and luciferase activity was evaluated using. Nano-Glo assay reagent. Results are expressed as the mean ± SD of three to four samples.

Supplementary. Fig. 3.

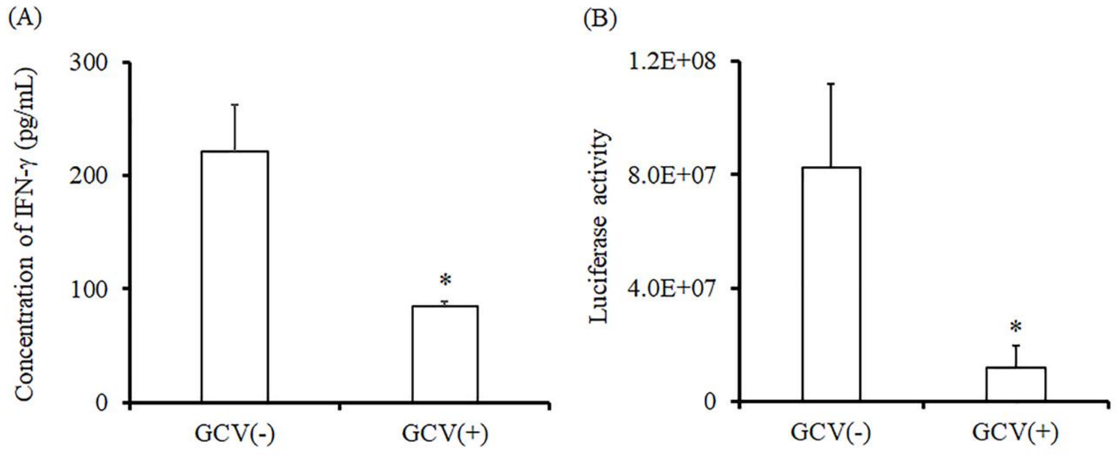

Supplementary. Fig. 3. IFN-γ concentration and luciferase activity in the skin of C3H10T1/2/HSVtk/IFN-γ cell- or C3H10T1/2/Nluc/HSVtk cell-transplanted mice. (A) C3H10T1/2/HSVtk/IFN-γ cells were transplanted into the back of BALB/c Slc-*nu/nu* mice. GCV was subcutaneously administered at a dose of 50 mg/kg soon after transplantation and every 12 h subsequently for ten consecutive days. The IFN-γ concentration in skin tissue was evaluated using an IFN-γ ELISA kit. Results are expressed as the mean ± SD of three to four samples. $*p < 0.05$, statistically significant differences observed in comparison with GCV (−) group. (B) C3H10T1/2/Nluc/HSVtk cells were transplanted into the back of BALB/c Slc-*nu/nu* mice. GCV was subcutaneously administered at a dose of 50 mg/kg soon after transplantation and every 12 h subsequently for ten consecutive days. The luciferase activity in skin tissue was evaluated using a Nano-Glo assay reagent. Results are expressed as the mean ± SD of three to four samples. $*p < 0.05$, statistically significant differences observed in comparison with GCV (−) group.

Supplementary. Fig. 4.

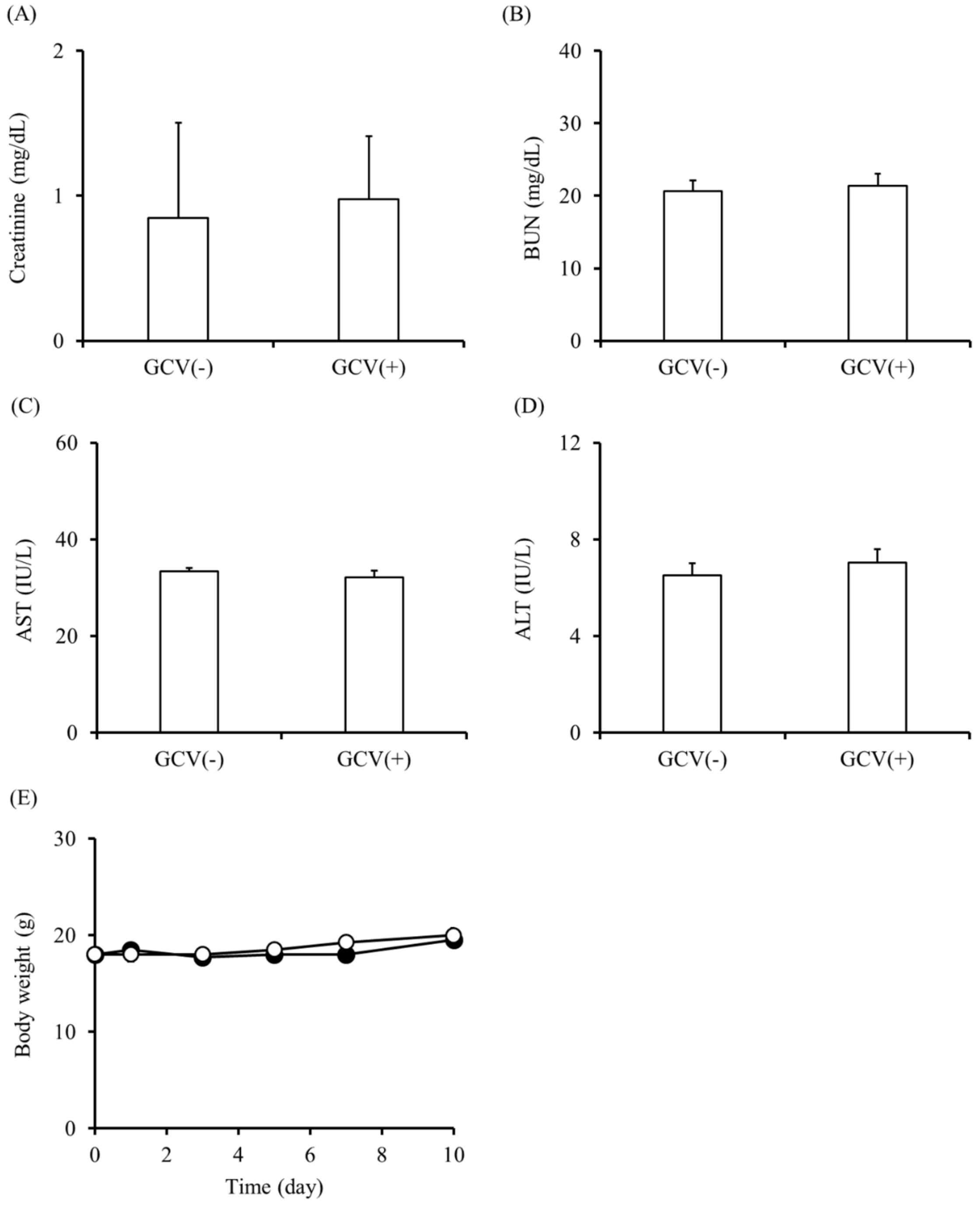

Supplementary. Fig. 4. Plasma levels of creatinine, BUN, AST and ALT, and body weight of mice. The blood was collected from the mice which C3H10T1/2/Nluc/HSVtk cells transplanted and GCV administration for 10 days. (A) creatinine, (B) BUN, (C) AST and (D) ALT activities and (E) body weight were measured. No GCV administration (white circle) and GCV administration (black circle) are indicated. Results are expressed

as the mean ± SD of four samples.
